# Clinician consensus on “Inappropriate” presentations to the Emergency Department in the Better Data, Better Planning (BDBP) census: a cross-sectional multi-centre study of emergency department utilisation in Ireland

**DOI:** 10.1186/s12913-023-09760-6

**Published:** 2023-09-18

**Authors:** Niamh M Cummins, Louise A Barry, Carrie Garavan, Collette Devlin, Gillian Corey, Fergal Cummins, Damien Ryan, Emma Wallace, Conor Deasy, Mary Flynn, Gerard McCarthy, BDBP Team, Tomas Barry, Martin Boyd, Des Fitzgerald, Peter Hayes, Gerry Lane, Geraldine McMahon, Rosa McNamara, Lisa McNamee, Anna Moore, Darragh O’Hare, Andrew O’Regan, Lorraine Reynolds, Rose Galvin

**Affiliations:** 1 School of Medicine, Faculty of Education and Health Sciences, SLÁINTE Research and Education Alliance in General Practice, Primary Healthcare and Public Health, University of Limerick, Limerick, Ireland; 2 Department of Paramedicine, Faculty of Medicine, Nursing and Health Sciences, Monash University, Melbourne, VIC Australia; 3 Ageing Research Centre, Health Research Institute, University of Limerick, Limerick, Ireland; 4 School of Allied Health, Faculty of Education and Health Sciences, University of Limerick, Limerick, Ireland; 5 Department of Nursing and Midwifery, Faculty of Education and Health Sciences, University of Limerick, Limerick, Ireland; 6 Emergency Department, ALERT Limerick EM Education Research Training, University Hospital Limerick, Limerick, Ireland; 7 Health Research Board Centre for Primary Care Research, Royal College of Surgeons in Ireland, Dublin, Ireland; 8 Emergency Department, Cork University Hospital, Cork, Ireland; 9 Emergency Medicine Programme, Royal College of Surgeons in Ireland, Dublin, Ireland

**Keywords:** Health Services Research, Healthcare Quality Improvement, Decision making, Emergency Department, General Practice

## Abstract

**Background:**

Utilisation of the Emergency Department (ED) for non-urgent care increases demand for services, therefore reducing inappropriate or avoidable attendances is an important area for intervention in prevention of ED crowding. This study aims to develop a consensus between clinicians across care settings about the “appropriateness” of attendances to the ED in Ireland.

**Methods:**

The Better Data, Better Planning study was a multi-centre, cross-sectional study investigating factors influencing ED utilisation in Ireland. Data was compiled in patient summary files which were assessed for measures of appropriateness by an academic General Practitioner (GP) and academic Emergency Medicine Consultant (EMC) National Panel. In cases where consensus was not reached charts were assessed by an Independent Review Panel (IRP). At each site all files were autonomously assessed by local GP-EMC panels.

**Results:**

The National Panel determined that 11% (GP) to 38% (EMC) of n = 306 lower acuity presentations could be treated by a GP within 24-48 h (k = 0.259; p < 0.001) and that 18% (GP) to 35% (EMC) of attendances could be considered “inappropriate” (k = 0.341; p < 0.001). For attendances deemed “appropriate” the admission rate was 47% compared to 0% for “inappropriate” attendees. There was no consensus on 45% of charts (n = 136). Subset analysis by the IRP determined that consensus for appropriate attendances ranged from 0 to 59% and for inappropriate attendances ranged from 0 to 29%. For the Local Panel review (n = 306) consensus on appropriateness ranged from 40 to 76% across ED sites.

**Conclusions:**

Multidisciplinary clinicians agree that “inappropriate” use of the ED in Ireland is an issue. However, obtaining consensus on appropriateness of attendance is challenging and there was a significant cohort of complex heterogenous presentations where agreement could not be reached by clinicians in this study. This research again demonstrates the complexity of ED crowding, the introduction of evidence-based care pathways targeting avoidable presentations may serve to alleviate the problem in our EDs.

**Supplementary Information:**

The online version contains supplementary material available at 10.1186/s12913-023-09760-6.

## Background

Emergency Department (ED) crowding is a global public health crisis which has been compounded by the COVID-19 pandemic [1]. Crowding occurs when the demand for ED services exceeds the resources available to provide urgent care to patients within an appropriate time frame [2]. The ED provides “rapid, high quality, continuously accessible, unscheduled care” for a wide range of acute illnesses and injuries and illnesses [3] but the primary purpose of the ED is to treat patients with potentially life-threatening illnesses and injuries. Therefore, ED crowding is a significant patient safety issue associated with increased morbidity and mortality [4]. The causes of ED crowding are multifactorial and relate to input, throughout and output factors. Input factors refer to the demand for ED services, throughput factors relate to the processes of evaluation and treatment within the ED, and output factors are associated with ED disposition [5].

An input factor which increases demand on ED services, but which is potentially avoidable, is utilisation of the ED for non-urgent care [6]. These attendances for non-urgent care, which could be adequately treated in other settings, such as primary care, are often referred to as “inappropriate” use of the ED. The difficulty in the ED is that urgent and non-urgent illnesses frequently manifest similarly therefore many low acuity symptoms can warrant attendance as potentially emergent conditions. It’s also the case that many non-urgent cases can still require advanced diagnostics, consultations, and even hospitalisation [7]. Therefore, for many patients these non-urgent presentations occur due to a gap in services and lack of alternative care pathways (ACP) in the community.

In Ireland, the Sláintecare Action Plan aims to improve population health by delivering the “right care, in the right place, at the right time, by the right team” [8]. The objective of this strategy is to shift the majority of care from the acute to the community setting. This is increasingly necessary because the acute hospital system in Ireland is under severe pressure, even prior to the COVID-19 pandemic the demand for emergency care had been increasing year on year [9]. This has culminated in the prevalent practicing of “Corridor Medicine” and a severe “Trolley Crisis” of patients who have been admitted to acute hospitals being treated on trollies while waiting for an available bed (also referred to as ED Boarding). Data compiled by the Irish Nurses and Midwives Organisation (INMO) indicates that over 70,275 patients were treated on trollies in Irish hospitals in 2021 [10]. In the community, primary care is under-resourced and recent data indicates that GP supply is an issue nationally, with an increased density of GPs required in rural and deprived areas [11].

A reduction of inappropriate or avoidable attendances is regarded as an important area for intervention by policymakers, who have focused on expanding access to primary care and improving triage systems in an effort to direct patients to the most appropriate care. A UK study involving 3,053 patients across 12 EDs investigating appropriateness of attendances, found that 15% of ED attendees were suitable for delayed management within 24 h by a GP in their surgery and a further 7% could have been treated by a GP working in the ED [12]. It is currently unknown how many patients presenting to the ED in Ireland could potentially utilise an ACP.

## Methods

### Aim

The aim of this research is to develop a consensus between health professionals across care settings about the “appropriateness” of attendances to the ED in Ireland utilising data from the Better Data, Better Planning (BDBP) Study.

### Design

The BDBP study was a multi-centre, cross-sectional study across urban and rural EDs (n = 5) in Ireland throughout 2020. The full methodology has previously been described [13].

### Setting

Following ethical approval, data were collected at each ED site over separate 24-h periods during the course of a year to account for diurnal and seasonal variation in attendance patterns. Participating hospitals included; Midlands Regional Hospital Tullamore (MRHT), University Hospital Limerick (UHL), St. Vincents University Hospital (SVUH), St. James University Hospital (SJUH) and University Hospital Kerry (UHK).

### Participants and procedure

BDBP was a Census study and at each site all adults presenting over a 24 h census period were eligible for inclusion. The inclusion criteria applied in the BDBP study were (A) Adult aged ≥ 18 years (B) Manchester Triage System (MTS) categories 2–5 and medically stable in relation to temperature, heart rate, respiratory rate, blood pressure, mental status and oxygenation (C) Patient has capacity and willingness to provide informed consent. Exclusion criteria include; (A) Scheduled admissions to the ED (B) Mental Health presentations (C) Patients with altered capacity due to drug or alcohol intoxication (D) Inability to communicate sufficiently in English to participate. Of the n = 601 patients attending the participating EDs over the 24 h Census period at each site a total of n = 306 (51%) were eligible for participation in the BDBP study [13]. For these patients clinical data were collected via electronic records and a questionnaire provided information on demographics, healthcare utilisation, service awareness and factors influencing the decision to attend the ED.

Following on from initial BDBP data collection, the Research Nurse performed a follow-up site visit to each ED to conduct a chart review for participants. Data were extracted from electronic patient records and an anonymised patient summary file was compiled (Supplementary Table S1) which included the following information; demographics, source of referral, current medications, social history, presenting complaint, duration of presenting complaint, Manchester triage category, vital signs at triage, patient’s level of self-reported pain and anxiety, investigations (e.g. blood sample, electrocardiogram; ECG, diagnostic imaging etc.), interventions (e.g. airway, suturing, splint etc.), medication administered (e.g. analgesic, fluids, antibiotic etc.) and referrals. These anonymised patient summary files were the sole unit of analysis for the consensus panel and the participating clinicians did not review the data from the patient questionnaires. The Research Nurse provided standardised training to all participating clinicians on the chart review and analysis.

### Measures of “Appropriateness” and rating scale analysis

The clinicians in this study were asked for responses to three questions;

- According to you, could the patient have been managed by a GP the same day or next day? Yes/No.
- According to you, was this patient’s ED visit an inappropriate use of ED resources? Yes/No.
- According to you, how appropriate was this ED visit? Rating Scale 0–10.

The “appropriateness” rating scale (0–10) was initially analysed using descriptive statistics. The scale was then coded as follows; inappropriate (0–3), neither appropriate or inappropriate (4–6) and appropriate (7–10). The proportion of attendances in each category was calculated and cross-tabulated for percentage agreement (consensus).

### Chart review

Patient Summary Files (n = 306) were initially assessed independently by a National Panel comprised of a Senior Academic GP and a Senior Academic Emergency Medicine Consultant (EMC) who both continue to practice clinically in their fields. The patient files for which there was no consensus (n = 136) between GP and EMC on “appropriateness” were subsequently assessed by an Independent Review Panel (IRP) of two additional EMCs (EMC2 and EMC3) and a Clinical Nurse Manager (CNM). No members of these autonomous panels had any knowledge of the sites where the data were collected and did not work or refer patients to any of the sites where data were collected. All of the Patient Summary files (n = 306) were then reviewed again at each ED site with a local GP and local EMC pair working independently.

### Data analysis

Data were entered into Excel (Microsoft, San Diego, CA), coded for analysis and analysed in SPSS (IBM SPSS Statistics Version 26, Armonk, NY). Variables were tested for normality using the Kolmogorov–Smirnov test. Variables are presented as mean (standard deviation; SD) or median (Interquartile Range; IQR), depending on distribution. Categorical data are presented as frequencies and percentages and the chi-square test was used to examine relationships between variables. Interobserver agreement was calculated using Cohen’s κ. The strength of agreement for κ are: values ≤ 0 indicate no agreement, none to slight 0.01–0.20, fair 0.21–0.40, moderate 0.41– 0.60, substantial 0.61–0.80, and 0.81–1.00 is almost perfect agreement. A single sample t-test was conducted in combination with a Bland Altman plot to illustrate levels of agreement graphically.

## Results

### National GP-EMC panel chart review (n = 306)

Three questions were asked of the National GP-EMC Panel with regard to “appropriateness” of patient attendances at the Emergency Department. In the BDBP Study, 11% of all attendances were considered to be suitable for management by a GP on the same day or the following day by the Academic GP, compared to 38% by the Academic EMC (Table 1). The level of consensus (% agreement) between the GP and EMC on management of patients by a GP within 24-48 h was 70% giving an inter-rater agreement of k = 0.259 (p < 0.0001).

A total of 18% of attendances were considered an inappropriate use of ED resources by the GP while in comparison the EMC considered 35% of attendances to be inappropriate. The level of consensus between GP and EMC on this was 73% with an inter-rater agreement of k = 0.341 (p < 0.001).

**Table 1:** National GP-EMC Panel Consensus and Inter-rater Agreement on Appropriate Attendances to the ED in the BDBP Study (n = 306)

Variable	General Practitioner (n, %)	Emergency Consultant (n, %)	Inter-rater Agreement (n, %) Kappa (IQR) P-value
**Management by GP in 24-48 h**	34, 11%	116, 38%	213, 70% 0.259 (0.166–0.351) p < 0.001
**Inappropriate Use of ED Resources**	56, 18%	108, 35%	223, 73% 0.341 (0.234–0.447) p < 0.001
**Appropriateness Rating Scale (1–10)**	Median 8 IQR 8–8 Range 2–10	Median 7 IQR 4–7 Range 1–10	0.144 (0.093–0.196) p < 0.001
**Inappropriate Attendance** ^**a**^ **Appropriateness Rating Scale (0–3)**	27, 9%	59, 19%	15, 5%
**Neither Appropriate nor Inappropriate Attendance** ^**a**^ **Appropriateness Rating Scale (4–6)**	8, 3%	91, 30%	3, < 1%
**Appropriate Attendance** ^**a**^ **Appropriateness Rating Scale (7–10)**	271, 89%	156, 51%	152, 50%

^a^Derived from trichotomisation of the Rating Scale

**Fig. 1:**
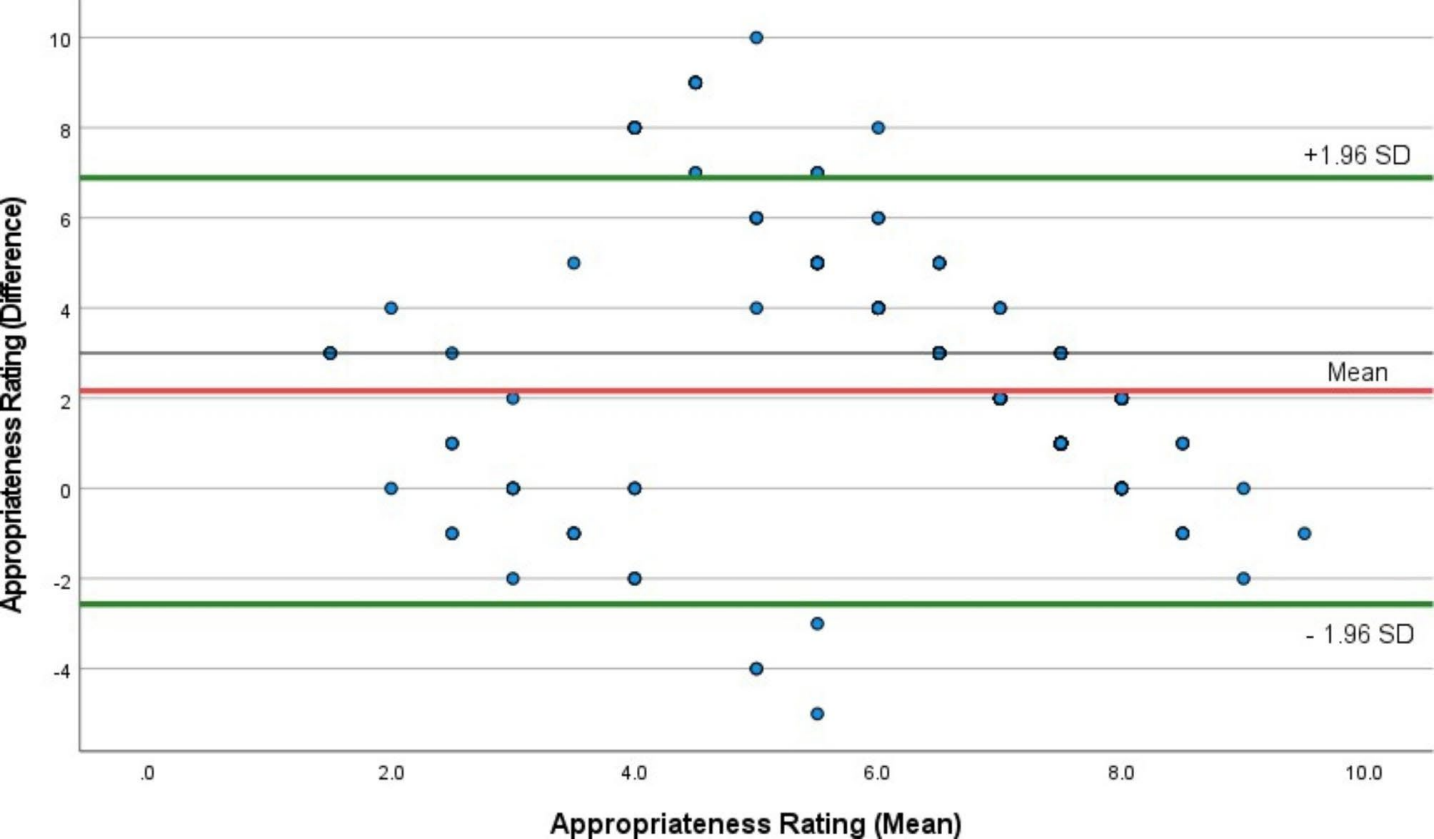
Bland Altman Plot of Appropriateness Rating by the National GP-EMC Panel for Attendance to the ED in the BDBP Study (n = 306)

The National GP-EMC Panel rated the appropriateness of all individual attendances to the ED during the BDBP Study (n = 306) on a Likert scale of 1–10. The Appropriateness Rating Scale assigned by the GP had a median of 8 and ranged from 2 to 10 while the median rating for the EMC was 7 and ranged from 1 to 10 (κ = 0.144; p < 0.001). The agreement between these measurements is illustrated in the Bland Altman Plot in Fig. 1 (B=-0.463; p < 0.001).

Based on the trichotomisation of this rating scale, the GP deemed 9% of attendances to be inappropriate compared to 19% by the EMC with an overall consensus rate of 5%. The consensus on appropriate attendances between the two clinicians was 50% (GP 89% vs. EMC 51%). Overall there was no consensus on 45% of charts (n = 136) reviewed by the National GP-EMC Panel and these patient charts were subsequently allocated to the IRP for additional analysis.

The demographic and clinical profile of patients in the BDBP Study and the self-reported factors influencing ED utilisation in Ireland have previously been described [13]. Details of the factors influencing ED attendance categorised by appropriateness of attendance, as determined by National GP-EMC Panel consensus are outlined in Table 2. Inappropriate attenders were less likely than appropriate attenders to consider their condition to be an emergency (27% vs. 55%; p < 0.05). Inappropriate attenders were also more likely than appropriate attenders to be unhappy with treatment by their GP (13% vs. 0%; p < 0.001), to attend because of ease of access (13% vs. 7% p < 0.05) or due to family recommendations (27% vs. 15% p < 0.05).

**Table 2:** Self-reported Factors influencing ED attendance among BDBP Participants (n = 306) categorised by Appropriateness of ED Attendance by National GP-EMC Panel Consensus

Self-Reported Reason for ED Attendance	“Inappropriate” Attendances (n = 15)	“Neutral” Attendance (n = 3)	“Appropriate” Attendances (n = 152)	No Consensus (n = 136)	P value
I saw my GP but was unhappy with the treatment	13%	0%	0%	2%	0.001
I’m unaware of other services to treat me for this problem	13%	67%	21%	35%	0.01
I consider this condition to be an emergency	27%	33%	55%	42%	0.05
The ED is the best place for my problem	60%	67%	74%	56%	0.05
It is easy for me to get to the ED	13%	33%	7%	3%	0.05
I attended the ED before and I was happy with it	0%	33%	8%	2%	0.05
My family told me to come to the ED	27%	0%	15%	6%	0.05

Details of the Demographic and Clinical Characteristics of BDBP Participants (n = 306) categorised by Appropriateness of ED Attendance by National GP-EMC Panel Consensus are outlined in Table 3. The number of patients deemed to be inappropriate attenders by both the GP and EM was low (n = 15) however both clinicians agreed that attendance were appropriate in n = 152 patients. Significant differences were observed across groups for a number of demographic and clinical variables. Inappropriate attenders were more likely than appropriate attenders not to have healthcare coverage (17% vs. 1%; p < 0.01) and to attend for musculoskeletal complaints (40% vs. 13%, p < 0.05). Significant differences were also observed across groups with regard to Triage, Length of Stay and Admission status. None of the patients deemed to be inappropriate attenders by both the GP and EMC were triaged as being “very urgent” and none of these patients were subsequently admitted. However, in cases where consensus was not reached across clinicians, 23% of attendances deemed inappropriate by the EMC were subsequently admitted, as were 20% of patients who were categorised by the EMC as neither appropriate nor inappropriate attendances.

**Table 3:** Demographic and Clinical Characteristics of BDBP Participants (n = 306) categorised by Appropriateness of ED Attendance by National GP-EMC Panel Consensus

Category	“Inappropriate” Attendances (n = 15)	“Neutral” Attendance (n = 3)	“Appropriate” Attendances (n = 152)	No Consensus (n = 136)	P value
**Gender** Female Male	7, 47% 8, 53%	1, 33% 2, 67%	76, 50% 76, 50%	69, 51% 67, 49%	0.933
**Age** Median IQR Range	44y 34–53 20–72	56y 44–59 31–61	54y 37–71 19–100	50y 31–67 18–92	0.213
**Civil Status** Partner/Married Separated/Divorced Widowed Single	8, 53% 2, 13% 1, 7% 4, 27%	1, 33% 2, 67% 0, 0% 0, 0%	93, 62% 9, 6% 11, 7% 37, 25%	77, 58% 8, 6% 13, 10% 35, 26%	0.05
**Healthcare Coverage** Public - No Cover Public - Medical Card Private Insurance	2, 17% 5, 42% 5, 42%	0, 0% 2, 100% 0, 0%	1, 1% 75, 58% 54, 42%	1, 1% 57, 54% 47, 45%	0.01
Presenting Complaint* Cardiovascular Gastroenterology Musculoskeletal Trauma	1, 7% 1, 7% 6, 40% 1, 7%	0, 0% 1, 33% 2, 67% 0, 0%	28, 19% 28, 19% 20, 13% 19, 13%	19, 14% 10, 7% 43, 32% 19, 14%	0.05
**Length of Stay in ED** Median IQR Range	2.57 h 0.37–5.44 0.04–7.21	4.27 h 2.70–8.32 1.13–12.37	6.07 h 3.39–10.34 0.02–19.49	4.41 2.25–8.01 0.11–67.12	0.001
**Triage** Very Urgent Urgent Standard Non-Urgent	0, 0% 4, 31% 8, 62% 1, 8%	0, 0% 0, 0% 3, 100% 0, 0%	43, 30% 87, 61% 12, 8% 1, 1%	15, 12% 69, 53% 45, 34% 2, 2%	0.001
**Admission** Yes No	0, 0% 15, 100%	0, 0% 3, 100%	63, 47% 71, 53%	28, 23% 94, 77%	0.001

^a^Most frequent categories listed for Presenting Complaint

^b^Individual cases of missing data were excluded from analysis, this occurred in a small number of cases E.g. Data was not traceable on hospital systems or in the event a participant chose not to respond to a question on the survey

### Independent review panel (IRP) chart review (n = 136)

As the level of non-consensus between the Academic GP and the Academic EMC was relatively high at 45% an Independent Chart Review was performed by two additional EM Consultants (EMC2 and EMC3) and a CNM on the n = 136 files for which consensus was not reached on “Appropriateness” in the full cohort. In this sample the GP deemed 9% of attendances to be inappropriate compared to 11% for the CNM, 32% for EMC1, 40% for EMC2 and 51% for EMC3 (Table 4).

**Table 4:** Comparison of “Appropriateness” of ED Attendance for n = 136 patients by the National Panel and Independent Review Panel (n = 136)

ED Attendance	GP	EMC1	EMC2	EMC3	CNM
**Appropriate**	119, 88%	4, 3%	46, 34%	32, 23%	88, 65%
**Neutral**	5, 4%	88, 65%	36, 27%	35, 26%	32, 24%
**Inappropriate**	12, 9%	44, 32%	54, 40%	69, 51%	15, 11%

With regard to consensus on “appropriateness” of attendances between clinicians the overall consensus rate ranged from 0 to 62% with the strongest agreement being between the GP and the CNM. For the EM Consultants the strongest agreement was between EMC2 and EMC3 with an overall consensus of 50% which would be considered to be “fair” according to Cohen’s kappa (ĸ=0.215, p ≤ 0.001). The “No Consensus” rate on appropriateness of attendances between clinician pairs ranged from 38 to 100%. The consensus rate for appropriate attendances specifically ranged from 0 to 59% and for inappropriate attendances ranged from 0 to 29%. The GP agreed most closely with EMC3 on inappropriateness with a consensus of 6%. For the EMCs the consensus on inappropriate attendances was highest between EMC2 and EMC3 at 29% (Supplementary Table S2).

### Local panel (GP-EMC) chart review (n = 306)

In the Local Panel analysis 32% of attendances were deemed suitable for management by a GP within 24-48 h, compared to 42% by the EMCs (Table 5). The consensus between the GPs and EMCs on management of patients by a GP within 24-48 h was 69% giving an inter-rater agreement of k = 0.330 (p < 0.001). Across sites the level of consensus ranged from 58% in UHL to 84% in SJUH. With regard to the use of ED resources, 45% of attendances were considered inappropriate by the GPs while in comparison the EMCs considered 28% of attendances to be inappropriate. The level of consensus between GPs and EMCs on this was 60% (k = 0.153; p < 0.001) and ranged from 22% in SVUH to 78% in SJUH. For the Local Panels the “Appropriateness” Rating Scale assigned by GPs and EMCs both had a median of 7 (IQR 5–8) and ranged from 0 to 10. The overall consensus on appropriateness based on the Rating scale was 55% and ranged from 40 to 76% across ED sites. Both the GPs and the EMCs overall deemed 14% of attendances to be inappropriate though this varied across sites from 0% in UHL and SVUH to 34% in MRHT for the GPs and from 11% in UHK to 21% in UHL for the EMCs.

**Table 5:** Local GP-EMC Panel Consensus and Inter-rater Agreement on Appropriate Attendances to the ED in the BDBP Study (n = 306)

Category	Variable	TOTAL n = 306	MRHT n = 41	UHL n = 57	SVUH n = 77	SJUH n = 67	UHK n = 64	Kappa IQR P-value
**Management** **By GP 24-48 h**	General Practitioner Emergency Consultant Consensus	32% 42% 69%	73% 34% 61%	11% 53% 58%	16% 39% 74%	30% 31% 84%	48% 53% 61%	0.330 0.230–0.442 p < 0.001
**Inappropriate** **Use of ED** **Resources**	General Practitioner Emergency Consultant Consensus	45% 28% 60%	39% 15% 76%	5% 28% 67%	91% 31% 22%	37% 24% 78%	38% 38% 69%	0.153 0.048–0.258 p < 0.01
**Appropriateness** **Rating Scale 1–10** Median (IQR)	General Practitioner Emergency Consultant	7 (5–8) 7 (5–8)	5 (3–7) 7 (5–8)	10 (8–10) 6 (4–7)	7 (6–8) 6 (4–9)	7 (5–8) 10 (5–10)	6 (3–10) 7 (5–8)	0.234 0.168-0.300 p < 0.001
**Appropriateness** **Consensus**	GP-EMC Consensus	55%	49%	40%	56%	76%	48%	0.276 0.187–0.364 p < 0.001
**Appropriate**	General Practitioner Emergency Consultant	63% 53%	32% 63%	95% 35%	73% 47%	58% 70%	48% 53%	-
**Neutral**	General Practitioner Emergency Consultant	23% 32%	34% 24%	5% 51%	27% 33%	27% 18%	22% 36%	-
**Inappropriate**	General Practitioner Emergency Consultant	14% 14%	34% 12%	0% 14%	0% 21%	15% 12%	30% 11%	-

## Discussion

The results of this study indicate that clinicians across healthcare settings agree that inappropriate use of the ED in Ireland is an issue, however obtaining consensus on what constitutes an inappropriate attendance to the ED remains challenging.

For lower acuity ED presentations in the BDBP study the National Panel determined that 11% (GP) to 38% (EMC) of n = 306 patients could be treated by a GP on the same day or the following day, compared to 32% (GP) and 42% (EMC) on the Local Panels for the same cohort of patients. In relation to utilisation of ED resources the National Panel deemed 18% (GP) to 35% (EMC) of attendances “inappropriate” in comparison to 45% (GP) and 28% (EMC) for the Local Panels. In Ireland access to urgent diagnostics and ACPs is currently subject to significant geographic variation nationally which may partially account for the differences between the National and Local panel findings. Differences in background and training of GPs and EMCs which would contribute to differing diagnoses across professions must also be acknowledged, however these findings also demonstrate that within the same profession accurate assessment is still challenging for individual patients, even among experienced clinicians.

A similar study in New Zealand also previously reported significant differences in the attitudes and perceptions of healthcare professionals involved in the referral, treatment, and admission of patients to the ED [14]. This is in agreement with the findings of a previous Irish study investigating healthcare providers’ perceptions of the appropriateness of ED attenders [15]. In that survey there was almost unanimous agreement among healthcare professionals that inappropriate attendance in Irish ED exists, with 98.8% of respondents stating that some patients attending the ED could be more appropriately treated elsewhere.

Patient self-assessment of illness severity has previously been reported to be a key driver for ED attendance [16]. An interesting finding of the BDBP study was the fact that inappropriate attenders were less likely than appropriate attenders to consider their condition to be an emergency (27% vs. 55%; p < 0.05). This suggests a level of awareness that the ED might not be the most appropriate place for their care, however it is known that some patients attend the ED due to gaps in other services, which raises the question of whether an attendance should be categorised as “inappropriate” based solely on clinical findings when other factors such as social issues are also relevant. Internationally there is no consensus among researchers and policymakers on a methodology for classification of ED attendances as appropriate or inappropriate [17] and as a consequence wide variability exists on the reported estimation of the prevalence (4.8‒90%) of inappropriate attendances [6]. The authors would also like to acknowledge the difficulties for patients with regard to utilisation of the term “appropriate” in regard to accessing emergency care and appreciate that the terminology is not ideal, despite being widely used in the medical literature.

Analysis of the appropriateness rating scale revealed that the National Panel (GP-EMC) agreed on the status of 169/306 presentations (55%). The panel deemed 151 presentations appropriate, 15 presentations inappropriate and agreed that a further 3 presentations were neither appropriate or inappropriate. Of the 151 presentations that were deemed appropriate, 91% were triaged as urgent or very urgent and 47% of patients were admitted to hospital whereas none of the presentations that were deemed inappropriate were admitted.

Patient charts for which there was no consensus (n = 136) between GP and EM on “appropriateness” were subsequently reviewed by an IRP of two additional EMCs (EMC2 and EMC3) and a CNM. With regard to consensus on “appropriateness” of attendances between clinicians the overall consensus rate ranged from 0 to 62% with the strongest agreement being between the GP and the CNM. For the EM Consultants the strongest agreement was between EMC2 and EMC3 with an overall consensus of 50% which would be considered to be “fair” according to Cohen’s kappa (ĸ=0.215, p ≤ 0.001). A total of 27% of these case presentations were admitted.

For presentations that are deemed appropriate by the consensus panel, the incidence of admission to hospital is 53%. The evidence base regarding strategies to reduce the incidence of hospital admission (e.g. Frailty Intervention Teams in the ED) should be further explored. Our recent randomised controlled trial (n = 353) demonstrates that a dedicated team of health and social care professionals significantly reduces the ED length of stay (median 12.1 versus 6.4 h, *p* < 0.001) and the incidence of hospital admission (55.9% versus 19.3%, *p* < 0.001) among lower acuity older adults presenting to the ED [18].

For attendances that are deemed inappropriate by the consensus panel, there are a number of successful ACPs underway in Ireland. Examples include the pilot of referrals from GPs to an ED Navigational Hub (ED NH) where referrals are screened by an appropriately trained healthcare professional with access to a senior clinical decision maker. A decision is taken if the ED NH can add value to the patient journey or if their needs are better met in another part of the integrated community or hospital continuum. More recently, integrated care teams for older adults in the community have engaged directly with GPs to take direct referrals of frail older adults for timely access to comprehensive geriatric assessment and intervention in the community. This could potentially be expanded and there may also potential for the establishment of a GP-EMC forum at regional sites. With regard to medical education it may also be beneficial for GP and EM specialist training schemes to offer specific opportunities to work in each other’s discipline.

Our findings demonstrate that there is a significant cohort of lower acuity ED presentations (45%) where agreement cannot be reached on appropriateness of attendance between healthcare providers across settings of care and within settings of care. The latter is evidenced by the lack of agreement among experienced EM clinicians where no consensus was reached on between 50 and 70% of case presentations. These represent the most challenging attendees as these are a heterogeneous group. A recent systematic review classified reduction interventions in the ED into three main types; primary care linkage, ED diversion, and cost-sharing or financial penalties [19]. Based on the findings of the BDBP Study, there is no simple solution to ED crowding in Ireland and it is likely that all of these approaches may be required to finally resolve the problem of the “Trolley Crisis” in our EDs.

### Limitations

The BDBP Study took place during 2020 and therefore the COVID-19 pandemic is a confounding variable for this research, in particular accessibility of GP services may have been impacted in the first wave of the pandemic. With regard to our admission data this refers only to the index visit for each patient and does not capture if the patient was subsequently admitted for the presenting complaint outside of the study period. Admission is also an imperfect standard for appropriateness of attendance at the ED, admission thresholds may differ across hospitals and there are other reasons for patients to be admitted acutely to hospital. These include the provision of further investigations and procedures and for these cases access via an alternate pathway would be more appropriate. A limitation of chart review analysis is that clinicians must make their decisions without the benefit of seeing the patient, which may have provided important additional information to inform their diagnosis and opinions on appropriateness of attendance. Also, details of the diagnostic tests ordered in the ED were included in the patient summary files, this information would not be available in the GP setting and is a further limitation of the chart review analysis. The BDBP Study was undertaken in 5 rural and urban EDs across Ireland and therefore the findings may not be entirely generalisable to international settings. The data presented in this paper relates to lower acuity clinical presentations only, which comprised approximately half of ED attendances during the study period. However, higher acuity presentations were deliberately excluded and so this could also be acknowledged as a strength as this meant the focus was solely on attendances that may be avoidable.

### Implications

Clinicians across healthcare settings agree that inappropriate use of the ED in Ireland is an issue, however obtaining consensus on what constitutes an inappropriate attendance to the ED remains challenging. The lack of agreement suggests that efforts to divert patients away from the ED may not be successful as clinicians outside of EDs do not agree with ED providers on the optimal destination for patients with complex heterogenous presentations. Therefore, defining the elusive consensus between health professions on what is deemed inappropriate could be a first step in reducing avoidable ED attendances. There is significant potential for the development of ACPs in the Irish setting. Additionally there is scope for improvements in education through participation in exchanges during specialist training schemes and collaboration through the establishment of a General Practice-Emergency Medicine Forum.

## Conclusions

Data from the BDBP study are essential to inform and guide the planning of urgent and emergency care services in the future. Based on our findings, there is no one solution that will solve ED crowding in Ireland. However, the introduction of evidence-based care pathways targeting appropriate and inappropriate ED attendees will serve to alleviate the problem of the Trolley Crisis in our EDs. For the clinically heterogeneous group where consensus wasn’t reached across healthcare professionals on appropriateness of ED attendance, further research is warranted to implement timely and appropriate care for this cohort.

### Electronic supplementary material

Below is the link to the electronic supplementary material.

Supplementary Material 1

## Data Availability

The datasets analysed during the current study are available from the corresponding author on reasonable request.

## Abbreviations

ACP: Alternative Care Pathway
BDBP: Better Data, Better Planning
CNM: Clinical Nurse Manager
ECG: Electrocardiogram
ED: Emergency Department
EMC: Emergency Medicine Consultant
GP: General Practitioner
HSE: Health Service Executive
INMO: Irish Nurses and Midwives Organisation
IQR: Interquartile Range
IRP: Independent Review Panel
UHK: University Hospital Kerry
MRHT: Midlands Regional Hospital Tullamore
MTS: Manchester Triage System
OPD: Out-Patient Department
UHL: University Hospital Limerick
SD: Standard Deviation
SVUH: St. Vincent’s’ Hospital Dublin
SJUH: St. James University Hospital Dublin
