# Correction: Clinician consensus on “Inappropriate” presentations to the Emergency Department in the Better Data, Better Planning (BDBP) census: a cross-sectional multi-centre study of emergency department utilisation in Ireland

**DOI:** 10.1186/s12913-023-10092-8

**Published:** 2023-10-09

**Authors:** Niamh M Cummins, Louise A. Barry, Carrie Garavan, Collette Devlin, Gillian Corey, Fergal Cummins, Damien Ryan, Emma Wallace, Conor Deasy, Mary Flynn, Gerard McCarthy, BDBP Team and Rose Galvin

**Affiliations:** 1 School of Medicine, Faculty of Education and Health Sciences, SLÁINTE Research and Education Alliance in General Practice, Primary Healthcare and Public Health, University of Limerick, Limerick, Ireland; 2 Department of Paramedicine, Faculty of Medicine, Nursing and Health Sciences, Monash University, Melbourne, VIC Australia; 3 Ageing Research Centre, Health Research Institute, University of Limerick, Limerick, Ireland; 4 School of Allied Health, Faculty of Education and Health Sciences, University of Limerick, Limerick, Ireland; 5 Department of Nursing and Midwifery, Faculty of Education and Health Sciences, University of Limerick, Limerick, Ireland; 6 Emergency Department, ALERT Limerick EM Education Research Training, University Hospital Limerick, Limerick, Ireland; 7 Health Research Board Centre for Primary Care Research, Royal College of Surgeons in Ireland, Dublin, Ireland; 8 Emergency Department, Cork University Hospital, Cork, Ireland; 9 Emergency Medicine Programme, Royal College of Surgeons in Ireland, Dublin, Ireland

**Correction to**: ***BMC Health Services Research*(2023) 23:1003**

10.1186/s12913-023-09760-6.

Following publication of the original article [1], the authors identified errors in the affiliation assignment, caused by a typesetting mistake: affiliation 3 (Ageing Research Centre, Health Research Institute, University of Limerick, Limerick, Ireland) was incorrectly omitted for the first author, Niamh Cummins.

This error is corrected in the affiliations list of this Correction article and the original article [1] has been updated.
