# Supplementary material for: Clinician consensus on “Inappropriate” presentations to the Emergency Department in the Better Data, Better Planning (BDBP) census: a cross-sectional multi-centre study of emergency department utilisation in Ireland
Source: BMC Health Serv Res. 2023 Sep 18;23:1003. doi: 10.1186/s12913-023-09760-6 (PMC10506270; doi:10.1186/s12913-023-09760-6)
Supplement: Supplementary file 1 — Supplementary Material 1 [file 12913_2023_9760_MOESM1_ESM.docx]

**Supplementary Table S1.** Patient Summary File

| **ED Study ID Code No.** | | |
| --- | --- | --- |
| **Patient Medical Information** | | |
| **Presenting time to ED** |  | |
| **Gender** |  | |
| **Age** |  | |
| **Presenting complaint** |  | |
| **Vital signs in Triage** |  |  |
| **Manchester triage category** |  | |
| **Patient report** on duration of presenting problem? *How long have you had this problem?* |  | |
| **Patient Anxiety perspective**  *How* ***worried*** *(10 being the most worried) would you say you are about the problem that brought you to the emergency department today?* |  | |
| **Patient Pain perspective**  *On a scale from 1 to 10, how would you rate your* ***pain level*** *(10 being the most severe) on arrival at the emergency department?* |  | |
| **Urinalysis** |  | |
| **Blood** work done (**Yes/No)** |  | |
| Haematology |  | |
| Biochemistry |  | |
| Serology/ Immunology |  | |
| Microbiology |  | |
| **ECG** |  | |
| Imaging |  | |
| ECHO (bedside) |  | |
| Advanced imaging studies (e.g. CT/MRI) |  | |
| Doppler/US |  | |
| Intravenous therapy (e.g. fluids, antibiotics, anti-pyretic, analgesics, anti-emetics). |  | |
| **Clinical interventions such as suturing, wound dressing, POP, traction, joint manipulation, splints, urinary catheter change or flush)** |  | |
| **Critical care intervention (e.g. airway assistance, inotropes)** |  | |
| **Referred to another speciality? (Yes/No)**  **Which speciality? (Surgery, medicine, orthopaedics, ENT, Ophthalmology** |  | |
| **Medications patient takes regularly if noted on ED record**  **(**name, dose and route of administration) |  | |
| **Social history** |  | |
| **Source of referral to ED** |  | |

**Supplementary Table S2.** Consensus on Appropriateness of ED Attendances by GP, CNM and EMC1 – EMC3 (n=136)

|  | **Overall**  **Consensus**  **n, %** | **Appropriate Consensus**  **n, %** | **Neutral Consensus**  **n, %** | **Inappropriate**  **Consensus**  **n, %** | **No**  **Consensus**  **n, %** | **Cohen’s Kappa** |
| --- | --- | --- | --- | --- | --- | --- |
| **GP-EM1** | 0, 0% | 0, 0% | 0, 0% | 0, 0% | 136, 100% | κ=-0.085  p≤0.0001 |
| **GP-EM2** | 48, 35% | 44, 32% | 0, 0% | 4, 3% | 88, 65% | ĸ=0.019  p≤0.609 |
| **GP-EM3** | 41, 30% | 30, 22% | 3, 2% | 8, 6% | 95, 70% | ĸ=0.056  p≤0.068 |
| **GP-CNM** | 83, 62% | 80, 59% | 2, 2% | 1, 1% | 53, 38% | ĸ=0.064  p≤0.214 |
| **EM1-EM2** | 53, 39% | 1, 1% | 27, 20% | 25, 18% | 83, 61% | ĸ=0.116  p≤0.05 |
| **EM1-EM3** | 41, 30% | 1, 1% | 18, 13% | 22, 16% | 95, 70% | ĸ=-0.055  p≤0.296 |
| **EM2-EM3** | 67, 50% | 17, 13% | 11, 8% | 39, 29% | 70, 50% | ĸ=0.215  p≤0.001 |
| **CNM-EM1** | 35, 25% | 3, 2% | 22, 16% | 10, 7% | 131, 75% | ĸ=0.064  p≤0.046 |
| **CNM-EM2** | 58, 43% | 40, 30% | 7, 5% | 11, 8% | 78, 57% | ĸ=0.151  p≤0.003 |
| **CNM-EM3** | 46, 34% | 29, 22% | 7, 5% | 10, 7% | 96, 66% | ĸ=0.099  p≤0.025 |
